# Supplementary material for: Isolated copper–tin atomic interfaces tuning electrocatalytic CO2 conversion
Source: Nat Commun. 2021 Mar 4;12:1449. doi: 10.1038/s41467-021-21750-y (PMC7933149; doi:10.1038/s41467-021-21750-y)
Supplement: Supplementary file 1 — Supplementary Information [file 41467_2021_21750_MOESM1_ESM.pdf]

Supplementary Information

Isolated copper-tin atomic interfaces tuning electrocatalytic CO_2 conversion

Wenhao Ren,[1,†] Xin Tan,[2,†] Jiangtao Qu,[3,4] Sesi Li,[5] Jiantao Li,[6] Xin Liu,[5] Simon P. Ringer,[3] Julie M. Cairney,[3,4] Kaixue Wang,[5] Sean C. Smith[2] and Chuan Zhao[1,]*

[1]School of Chemistry, University of New South Wales, Sydney, New South Wales 2052, Australia. [2]Integrated Materials Design Laboratory, Department of Applied Mathematics, Research School of Physics, The Australian National University Canberra, ACT 2601 (Australia). [3]Aerospace, Mechanical and Mechatronic Engineering. [4]Australian Centre for Microscopy and Microanalysis, the University of Sydney, 2006, NSW, Australia. [5]School of Chemistry and Chemical Engineering, Shanghai Jiao Tong University, Shanghai 200240, China. [6]State Key Laboratory of Advanced Technology for Materials Synthesis and Processing, Wuhan University of Technology, Wuhan 430070, China. [†]These authors contributed equally to this work. Correspondence and requests for materials should be addressed to C.Z. (chuan.zhao@unsw.edu.au).

Experimental Section:

Quantification of gas and liquid products. The products and Faradic efficiency (FE) of CO_2 reduction were measured using chronoamperometry at each fixed potential. The gaseous products were quantified by a gas chromatograph (GC) equipped with a flame ionization detector (FID) for CO and CH_4 and a thermal conductivity detector (TCD) for H_2 quantification. Ultrapure Ar (99.999%) was used as the carrier gas. The average flow rate of CO_2 was controlled at 20 ml min^{-1} by a Cole-Parmer mass flow meter. GC was calibrated using standard gas mixtures under standard conditions (1 atm, 298 K). Liquid products were analysed on a 400 MHz nuclear magnetic resonance spectrometer (NMR, Bruker Avance III HD). A 0.5 ml of product-containing electrolyte was syringed out from the cathodic compartment after electrolysis. It was mixed with 0.1 ml of internal standard of diluted dimethyl sulfoxide (DMSO, Sigma-Aldrich, >99.9%) and 0.1 ml of D_2O (Cambridge Isotope Lab, 99.9%), and then transferred to an NMR sample tube. The calibration curve of formate was made by using sodium formate (Sigma-Aldrich, >99.998%) with different concentrations together with the internal standard and D_2O.

DFT calculations. All of the spin-polarized DFT calculations were performed using the VASP program,[1-3] which uses a plane-wave basis set and a projector augmented wave method (PAW) for the treatment of core electrons.[2] The Perdew, Burke, and Ernzerhof exchange-correlation functional within a generalized gradient approximation (GGA-PBE)[4] was used in our calculations, and the van der Waals (vdW) correction proposed by Grimme (DFT-D3)[5] was employed due to its good description of long-range vdW interactions. For the expansion of wavefunctions over the plane-wave basis set, a converged cutoff was set to 450 eV. Spin-polarization effect and dipole correction were considered in all cases.

Figure 5a shows the theoretical models of the studied systems. The structural models of pure Cu, Cu-Sn surface alloy, Cu-Sn bulk alloy, and Cu-Sn core-shell bulk alloy are constructed as 2×4 periodic supercells, which contains six atomic layers with the bottom three layers fixed in their respective bulk positions and all the other atoms

fully relaxed. Here, we considered stepped facets (211) in our models, which have been found to be generally more active for CO_2RR than flat terrace sites. In order to simulate 1% or 3% Sn-doped Cu, a Sn atom replaces a surface Cu atom, which shows much smaller substitution energy rather than in layers below the surface.[6] The vacuum space was set to larger than 15 Å in the z-direction to avoid interactions between periodic images. In geometry optimizations, all the structures were relaxed up to the residual atomic forces smaller than 0.005 eV/Å, and the total energy was converged to 10^{-5} eV. The Brillouin zone integration was performed on the (3×3×1) Monkhorst–Pack k-point mesh.[7]

For CO_2RR on various catalysts, we considered different reaction mechanisms of CO_2RR to formic acid and CO, which have been proposed for CO_2RR on Cu-Sn alloys.[8]

Formic Acid:

$CO_2 + H^+ + e^- + {}^* \rightarrow OCHO^*$ (1)

$OCHO^* + H^+ + e^- \rightarrow HCOOH + {}^*$ (2)

$CO_2 + H^+ + e^- + {}^* \rightarrow COOH^*$ (3)

$COOH^* + H^+ + e^- \rightarrow HCOOH + {}^*$ (4)

Carbon monoxide:

$COOH^* + H^+ + e^- \rightarrow CO^* + H_2O$ (5)

$CO^* \rightarrow CO + {}^*$ (6)

The free energy changes at each electrochemical step involving a proton-electron transfer were computed based on computational hydrogen electrode (CHE) model,[9] in which the free energy of $(\mathrm{H}^+ + e^-)$ equals to $\frac{1}{2}\mathrm{H}_2(g)$ for standard hydrogen electrode (SHE). The free energy of adsorbates and non-adsorbed gas-phase molecules is calculated as $G = E_{elec} + E_{ZPE} + \int C_P dT - TS$, where E_{elec} is the electronic energy calculated by DFT; E_{ZPE} is the zero point energy (ZPE), C_P is heat capacity, T is temperature and S is entropy. Here, the correction terms are present in Table S3, which are from previous literature.[9,10] Additionally, a correction of –0.32 and +0.26 eV for non-adsorbed gas-phase CO and HCOOH molecules have

to be made due to the use of PBE functional.[8] The solvation effects have been included for $COOH^*$ and CO^* by stabilizing 0.25 and 0.10 eV, respectively.[9,10]

The overall hydrogen evolution reaction (HER) mechanism is evaluated with a three-state diagram consisting of an initial H^+ state, an intermediate H^* state, and 1/2 H_2 as the final product. The free energy of H^* (ΔG_{H^*}) is proven to be a key descriptor to characterize the HER activity of the electrocatalyst. An electrocatalyst with a positive value leads to low kinetics of adsorption of hydrogen, while a catalyst with a negative value leads to low kinetics of release of hydrogen molecule.[11] The optimum value of $|\Delta G_{H^*}|$ should be zero; for instance, this value for the well-known highly efficient Pt catalyst is near-zero as $|\Delta G_{H^*}| \approx 0.09$ eV.[11] The ΔG_{H^*} is calculated as[11]

$$\Delta G_{H^*} = \Delta E_{H^*} + \Delta E_{ZPE} - T\Delta S_H \qquad (7)$$

where ΔE_{H^*} is the binding energy of adsorbed hydrogen, and ΔE_{ZPE} and ΔS_H are the difference in ZPE and entropy between the adsorbed hydrogen and hydrogen in the gas phase, respectively. As the contribution from the vibrational entropy of hydrogen in the adsorbed state is negligibly small, the entropy of hydrogen adsorption is $\Delta S_H \approx -\frac{1}{2} S_{H_2}$, where S_{H_2} is the entropy of H_2 in the gas phase at the standard conditions. Therefore, Eq. (7) can be rewritten as[11]

$$\Delta G_{H^*} = \Delta E_{H^*} + 0.24 \text{ eV} \qquad (8)$$

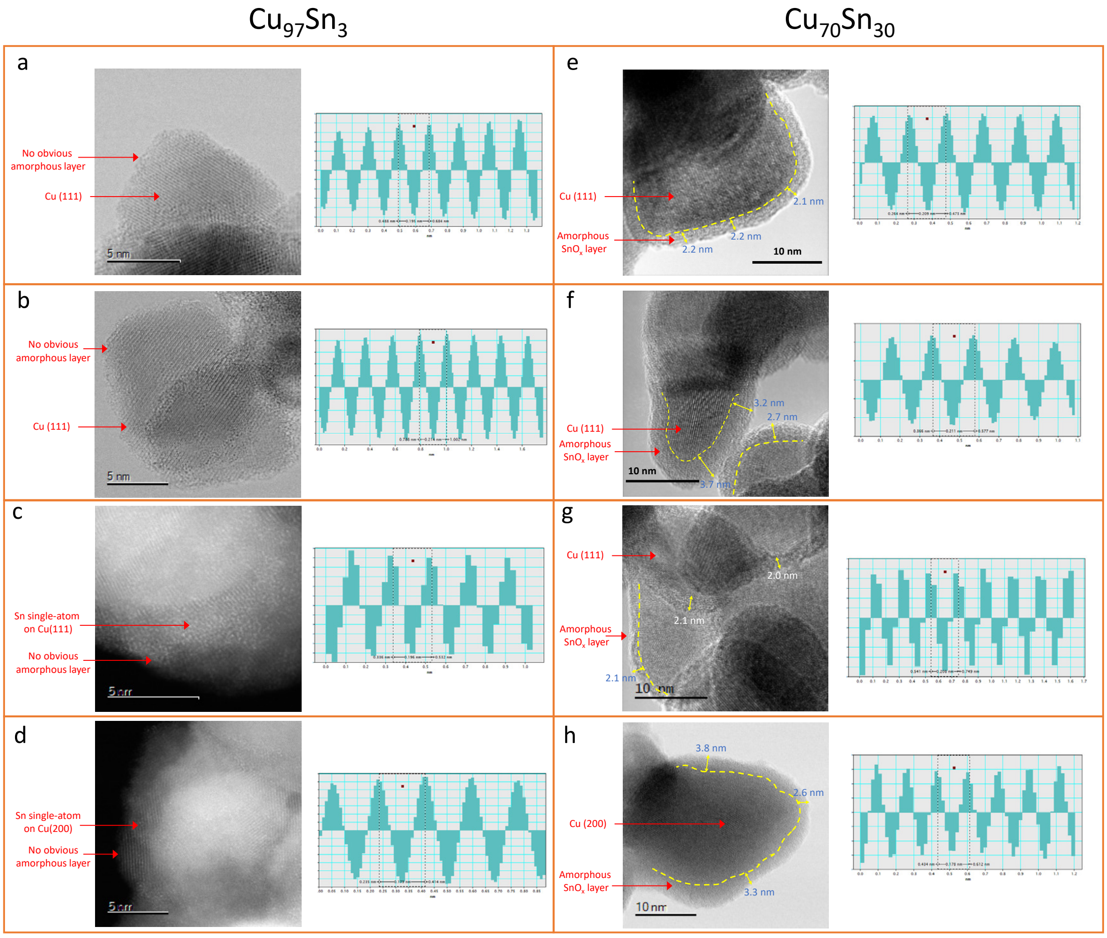

Supplementary Figure 1 | TEM characterizations of $Cu_{97}Sn_3$ and $Cu_{70}Sn_{30}$. (**a-d**) The HRTEM and HAADF-STEM images of $Cu_{97}Sn_3$ with the spacing analysis of Cu lattice. (**e-h**) The HRTEM images of $Cu_{70}Sn_{30}$ with the spacing analysis of Cu lattice. There is a relatively thick amorphous SnO_x shell (2.0-3.8 nm) out of Cu nanoparticles on $Cu_{70}Sn_{30}$.

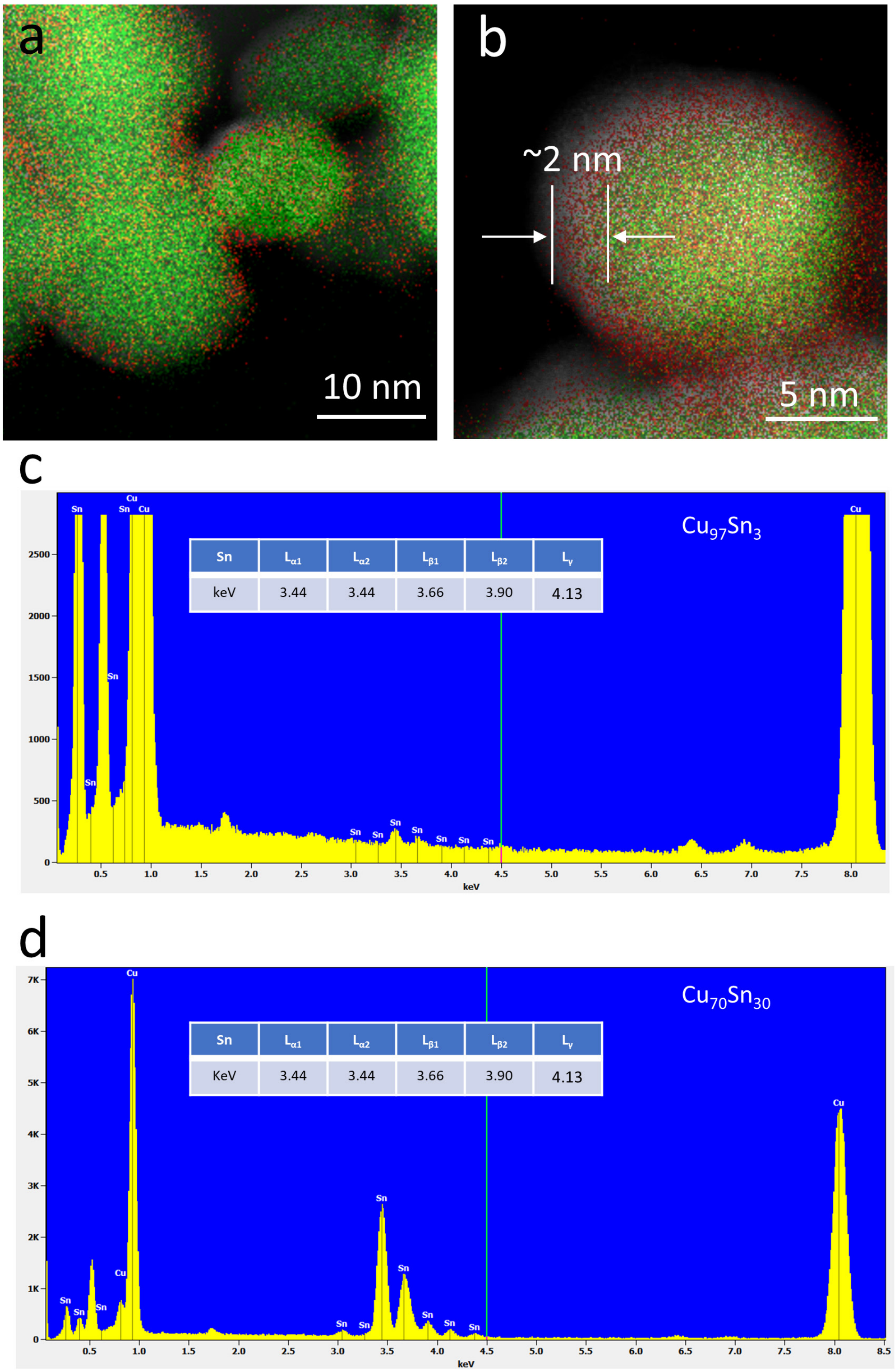

Supplementary Figure 2 | EDS analysis of $Cu_{97}Sn_3$ and $Cu_{70}Sn_{30}$. (**a,b**) The overlapped EDX mapping of $Cu_{97}Sn_3$ and $Cu_{70}Sn_{30}$ based on Fig. 1b,c, respectively. Cu (green) and Sn (red). (**c,d**) The corresponding mass spectrum analysis.

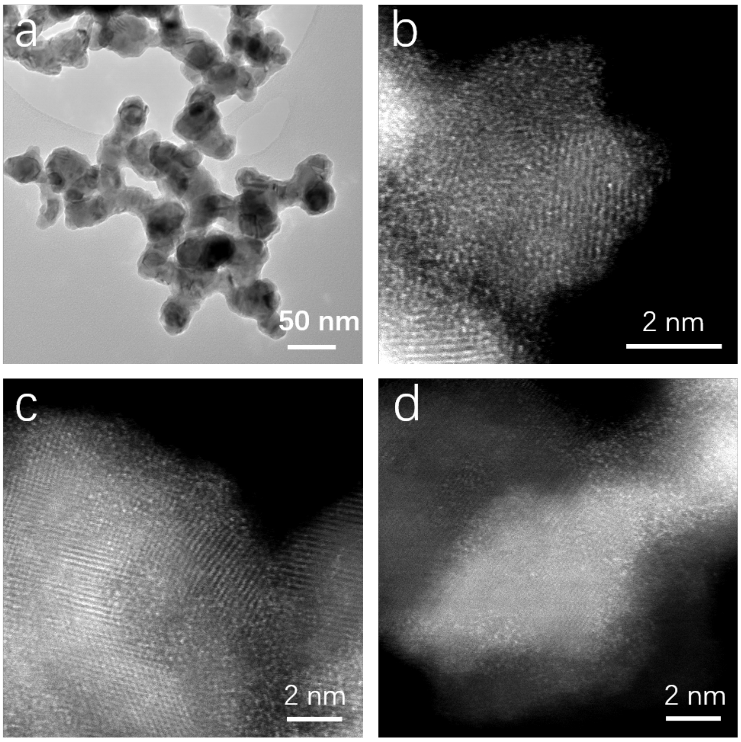

Supplementary Figure 3 | TEM images of $Cu_{97}Sn_3$. (**a**) Typical TEM image $Cu_{97}Sn_3$ nanoparticles. (**b-d**) HAADF-STEM images showing atomically dispersed Sn on the Cu host surface.

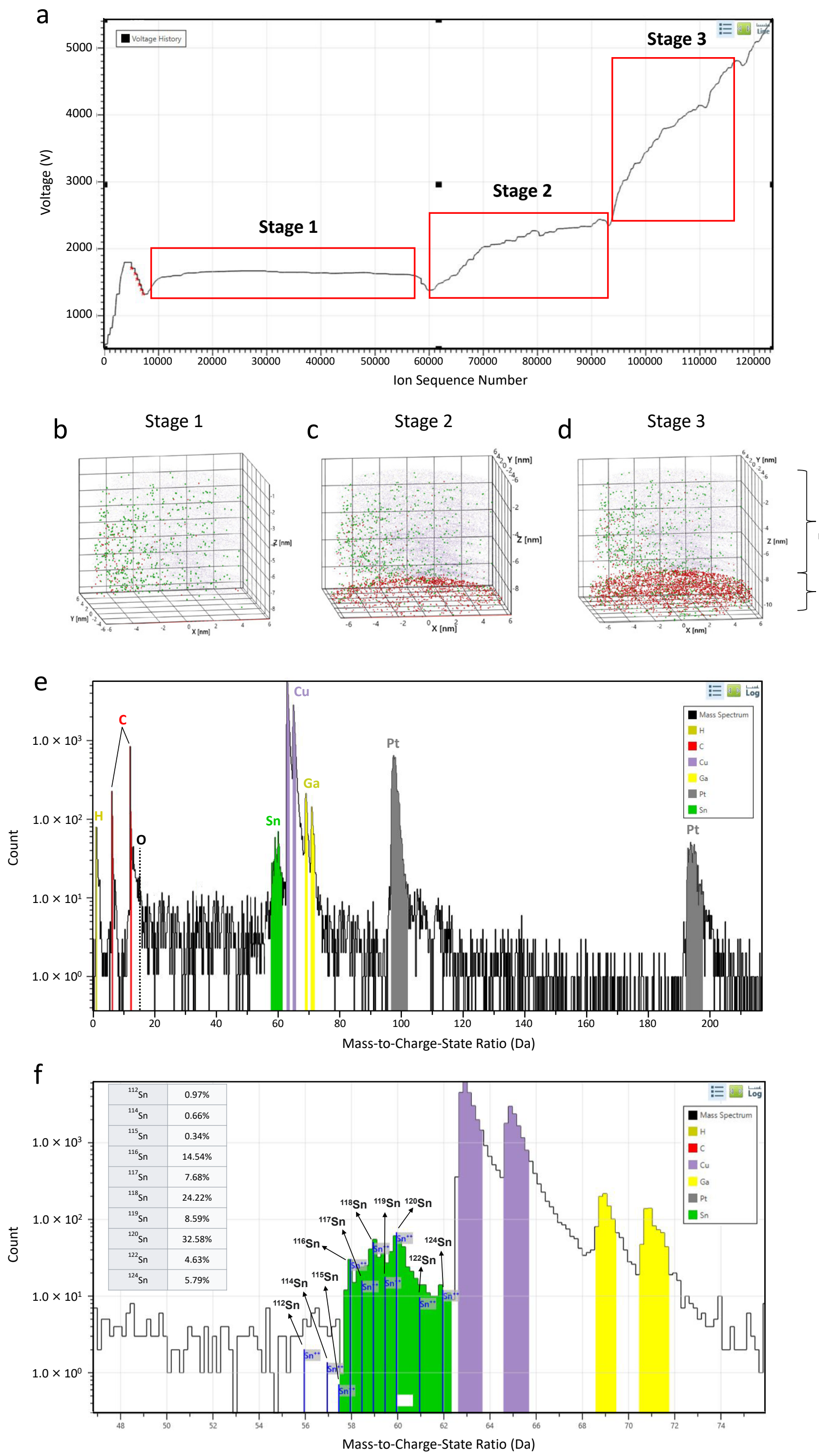

Supplementary Figure 4 | Atom probe tomography analysis of $Cu_{97}Sn_3$ specimen. (**a**) Voltage curves. 3D tomography at the end of Stage 1 (**b**), Stage 2 (**c**), and Stage 3 (**d**). (**e**) The full-range mass to charge spectrum with rebin-treatment to enhance the signal to background ratio. (**f**) The detailed Sn isotopes analysis. Inset is the natural abundance of main isotopes of tin and the ratio is demonstrated as the blue lines, which is in good agreement with the experimental results.

The whole APT experiment includes three stages. During Stage 1, the $Cu_{97}Sn_3$ alloy was successfully evaporated by applying electric pulses at the voltage of 1,600 V, which is a common voltage observed for alloy samples (Supplementary Fig. 4a). After the stable evaporation, we obtained the tomography of a single nanoparticle (Supplementary Fig. 4b). Then, the voltage started to increase in Stage 2 because of the detection of the C (Pt) signals (Supplementary Fig. 4c). The C (Pt) comes from our specimen preparation process, during which we incorporated the nanoparticles into a layer of electron-beam-assisted deposition of $(C_5H_4)CH_3Pt(CH_3)_3$. This deposition can fill the interspace of each nanoparticle and make the tip compacted. However, the carbon needs higher energy to be evaporated so that the voltage gradually increases.[12] As the experiment continued to run, the carbon layer became thicker and the voltage even increased to above 5,000 V after Stage 3 (Supplementary Fig. 4d). In this case, the APT experiment would automatically end, and we cannot obtain more tomography of nanoparticles.

The full-range mass-to-charge spectrum is shown in Supplementary Fig. 4e. Our specimen preparation method use gas-injection-system as adhesive to bond the nanoparticle onto the Mo post, followed by the $(C_5H_4)CH_3Pt(CH_3)_3$ deposition and a Ga source FIB annular milling.[13] As a result, C, Pt, H, and Ga are the primary contamination introduced to the sample. Note that all the elements detected in the spectrum (H, C, Sn, Cu, Ga and Pt) have no overlapping owing to their different mass-to-charge-state ratio. Besides, the overall Sn content is more than 1% in the specimen and the surface content can reach up to ~8%, which is far beyond the detection limit of APT technique (ppm elemental detection capacity).[14] Another strong evidence is that the mass spectrum of Sn matches very well to the corresponding isotopes (Supplementary Fig. 4f). Based on the above analysis, we can firmly claim that the signal peak at ~60 (Da) originating from Sn only, rather than impurities or noise.

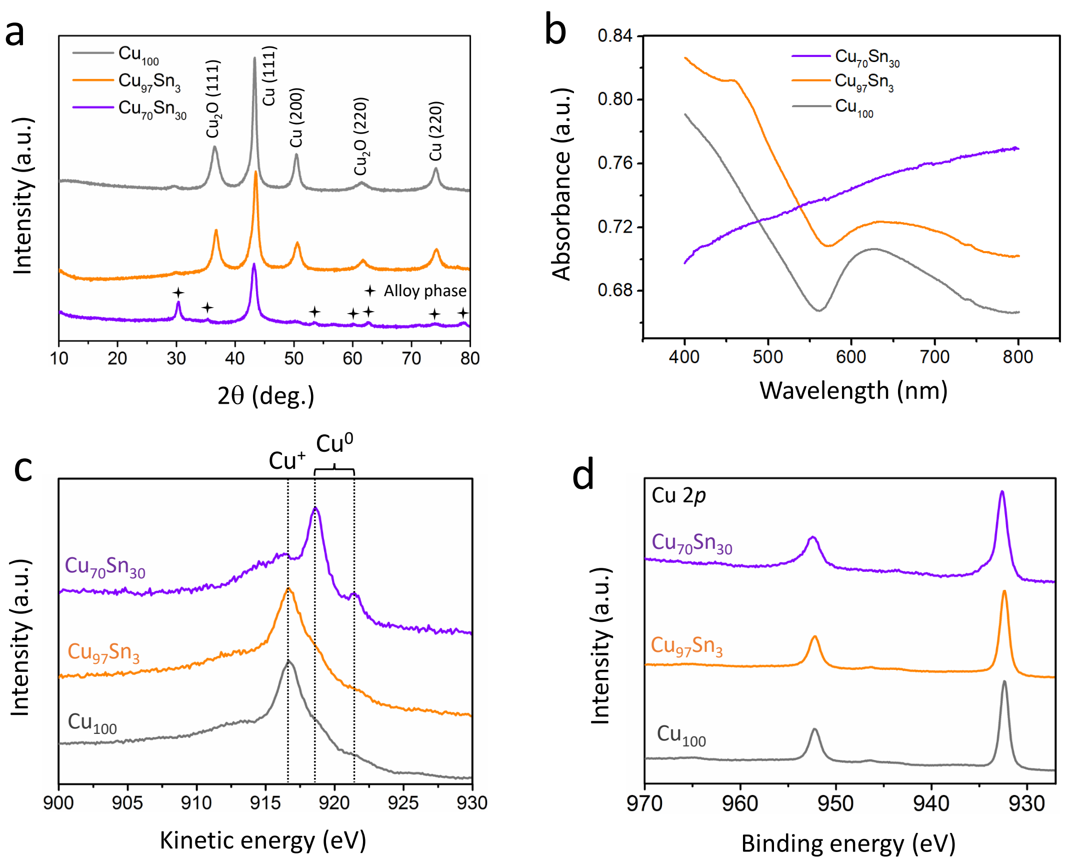

Supplementary Figure 5 | Structural characterizations of Cu_{100}, $Cu_{97}Sn_3$ and $Cu_{70}Sn_{30}$. (**a**) XRD patterns. (**b**) UV-vis spectroscopy. (**c**) Cu LMM Auger spectra and (**d**) XPS of Cu 2*p* spectra.

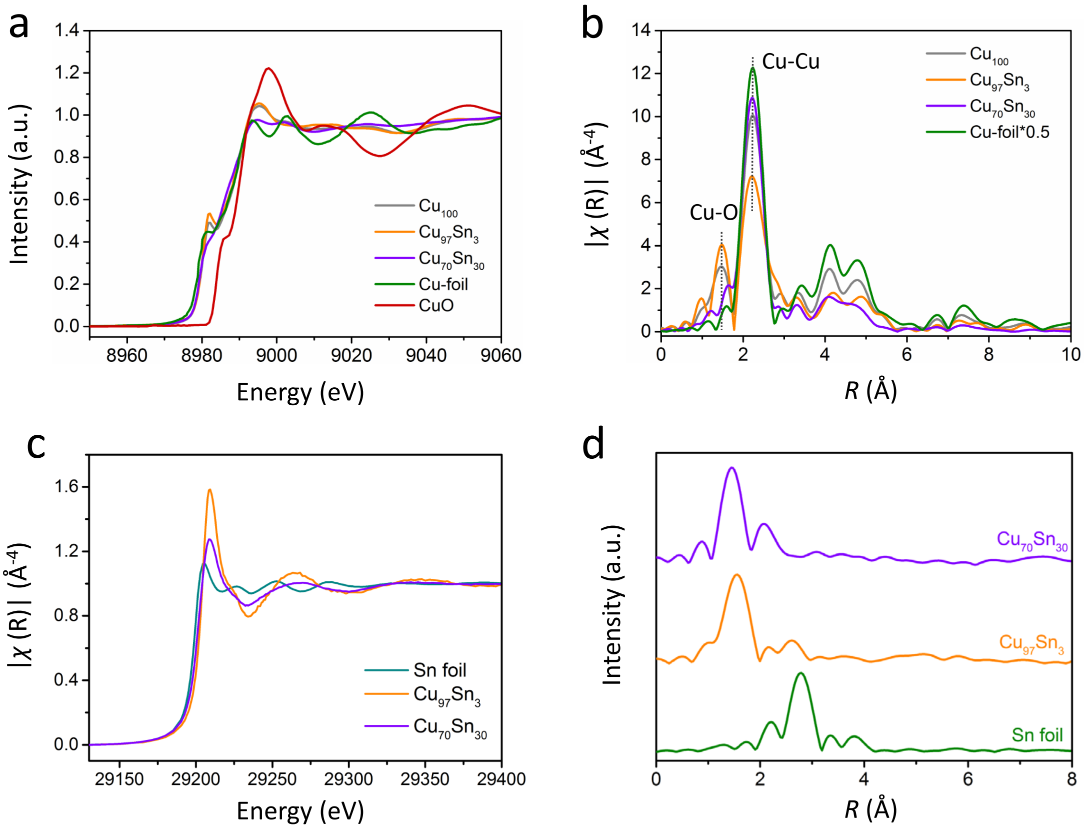

Supplementary Figure 6 | X-ray absorption spectroscopy. (**a**) Cu K-edge XANES spectra of Cu_{100}, $Cu_{97}Sn_3$ and $Cu_{70}Sn_{30}$ with the comparison of standard CuO and Cu-foil. (**b**) The plotted FT-EXAFS spectra obtained from Cu K-edge absorption spectra. (**c**) Sn K-edge XANES spectra of $Cu_{97}Sn_3$ and $Cu_{70}Sn_{30}$ with the comparison of standard Sn-foil. (**d**) The plotted FT-EXAFS spectra obtained from Sn K-edge absorption spectra. As shown in Figure S6c, the near-edge positions of $Cu_{97}Sn_3$ and $Cu_{70}Sn_{30}$ catalysts are higher than that of Sn foil, indicating the oxidation of Sn in the samples. This is also supported by the Fourier-transformed k^3-weighted spectra of the samples, where the peaks at 1.4 Å correspond to Sn-O coordination shells.

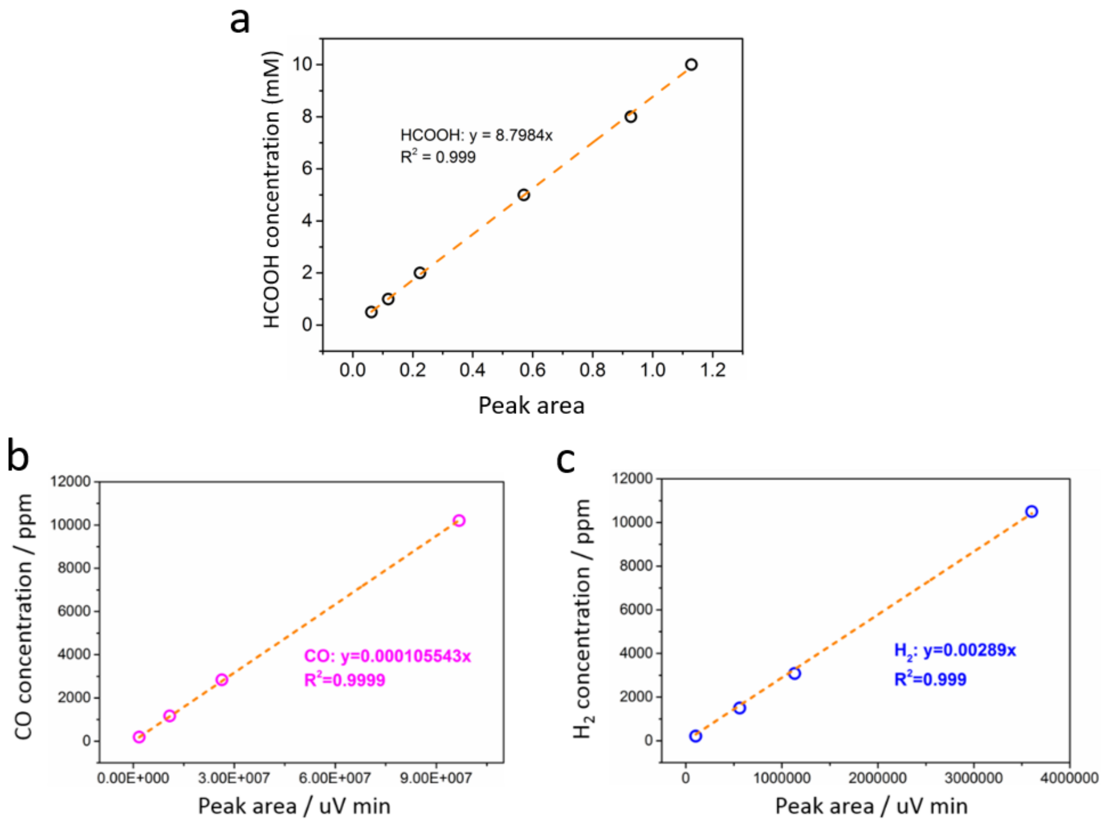

Supplementary Figure 7 | Calibration curves for different products. (**a**) NMR calibration curves for HCOOH. (**b,c**) GC calibration curves for CO and H_2, respectively.

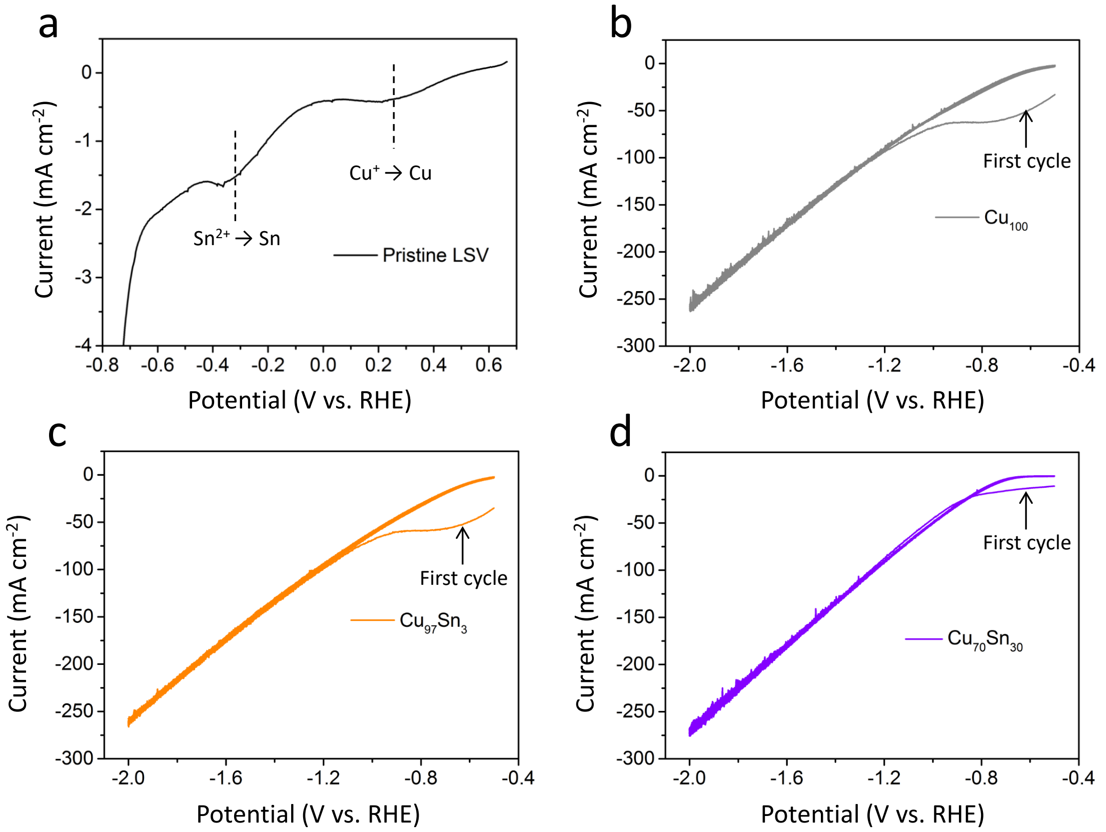

Supplementary Figure 8 | CV pretreatment. (**a**) Pristine LSV curves of $Cu_{70}Sn_{30}$. (**b-d**) CV pretreatment from -0.5 to -2.0 V vs. RHE for 10 cycles at 50 mV s^{-1}.

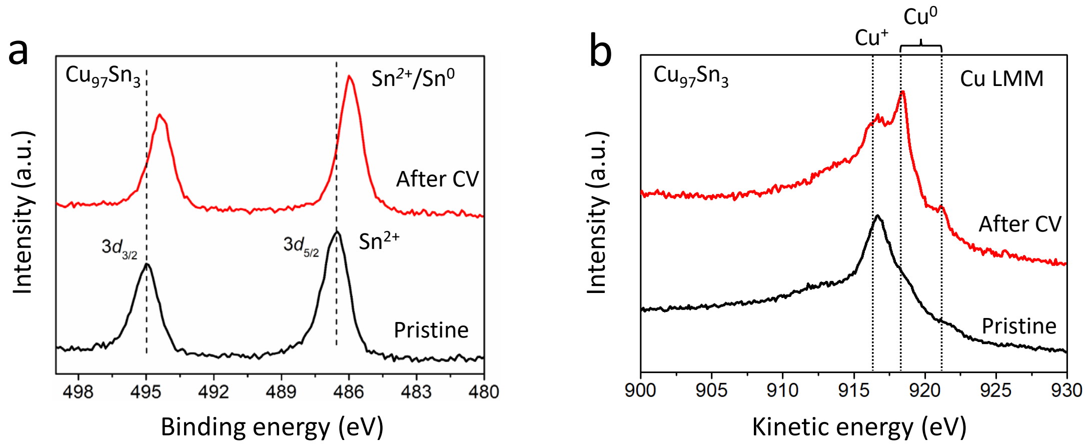

Supplementary Figure 9 | Structural analysis of $Cu_{97}Sn_3$ before and after CV treatment. (**a**) XPS of Sn 3*d* spectra. (**b**) Cu LMM Auger spectra. *Ex-situ* Sn 3*d* XPS and Cu LMM Auger spectra on $Sn_{97}Sn_3$ confirm the reduction of Sn^{2+} to Sn^0 and Cu^+ to Cu^0 after the CV pretreatment. The residual oxides can be attributed to the inevitable oxidation during sample delivery and test. It is expected that most of the Cu and Sn species are reduced to the metallic state after the CV treatment, according to their standard reduction potential ($Sn^{2+} + 2e^- \rightleftharpoons Sn$ at -0.13 vs. SHE, $Cu^+ + e^- \rightleftharpoons Cu$ at +0.52 vs. SHE).[6,8,15]

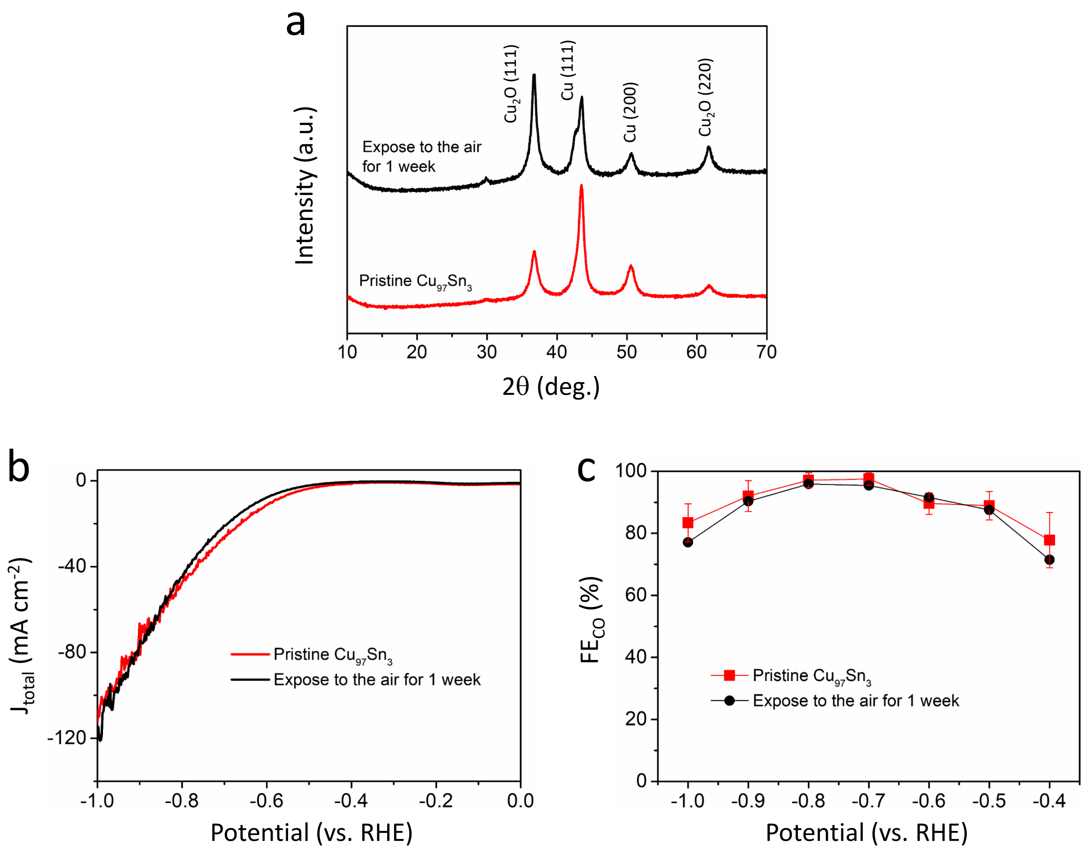

Supplementary Figure 10 | CO_2RR assessment of $Cu_{97}Sn_3$ after exposure to the air for 1 week. (**a**) XRD analysis. (**b**) LSV comparison. (**c**) FE_{CO} from -0.4 to -1.0 V vs RHE. To further investigate the influence of the surface oxide layer, the as-synthesized $Cu_{97}Sn_3$ sample is exposed to the air and aged for 1 week before CO_2RR testing. As shown in XRD analysis, the $Cu_{97}Sn_3$ catalyst is further oxidized after continuous exposure to the air. While, after the CV pretreatment, the aged $Cu_{97}Sn_3$ show very close LSV curves and similar FE_{CO} compared with pristine $Cu_{97}Sn_3$ for CO_2 electrolysis, indicative of the similar CO_2RR reactivity and the negligible effect of surface oxides after CV pretreatment.

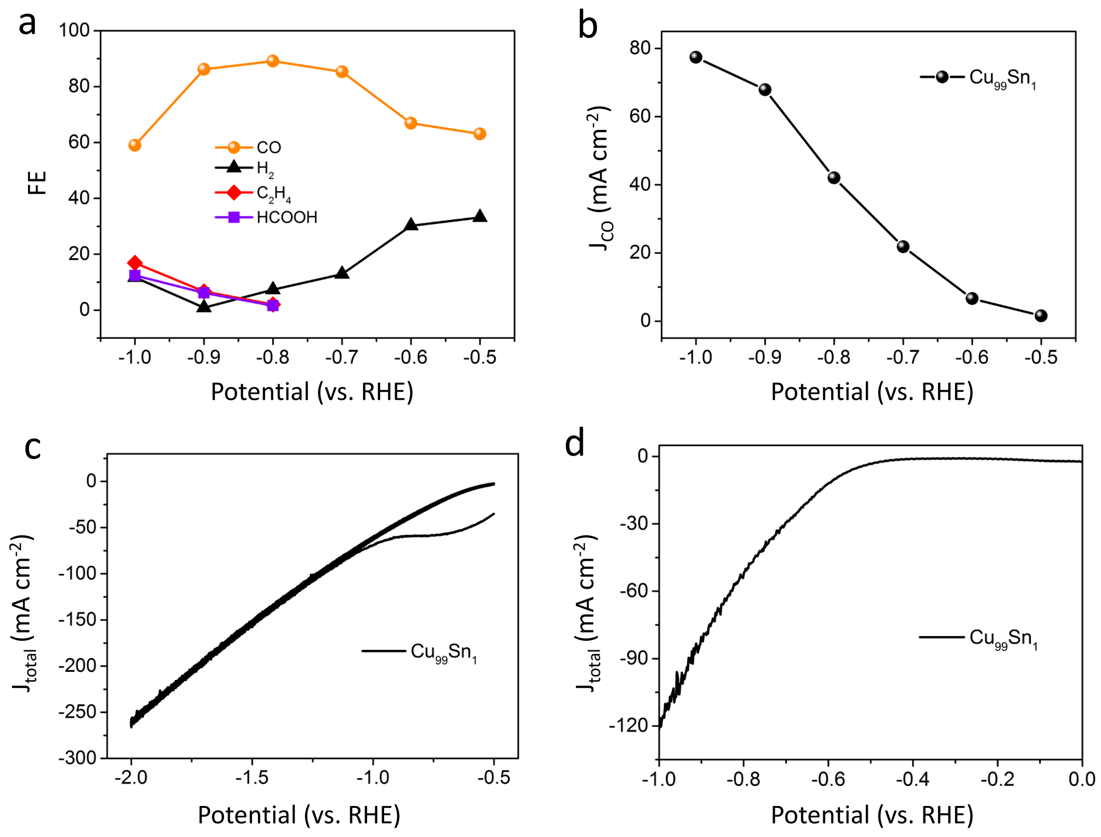

Supplementary Figure 11 | CO_2RR performance of $Cu_{99}Sn_1$. (**a**) Potential dependence of Faradaic efficiencies from -0.5 to -1.0 vs. RHE. (**b**) Potential dependence of j_{co}. (**c**) CV pretreatment at 50 mV s^{-1}. (**d**) LSV curves from 0 to -1.0 V vs. RHE.

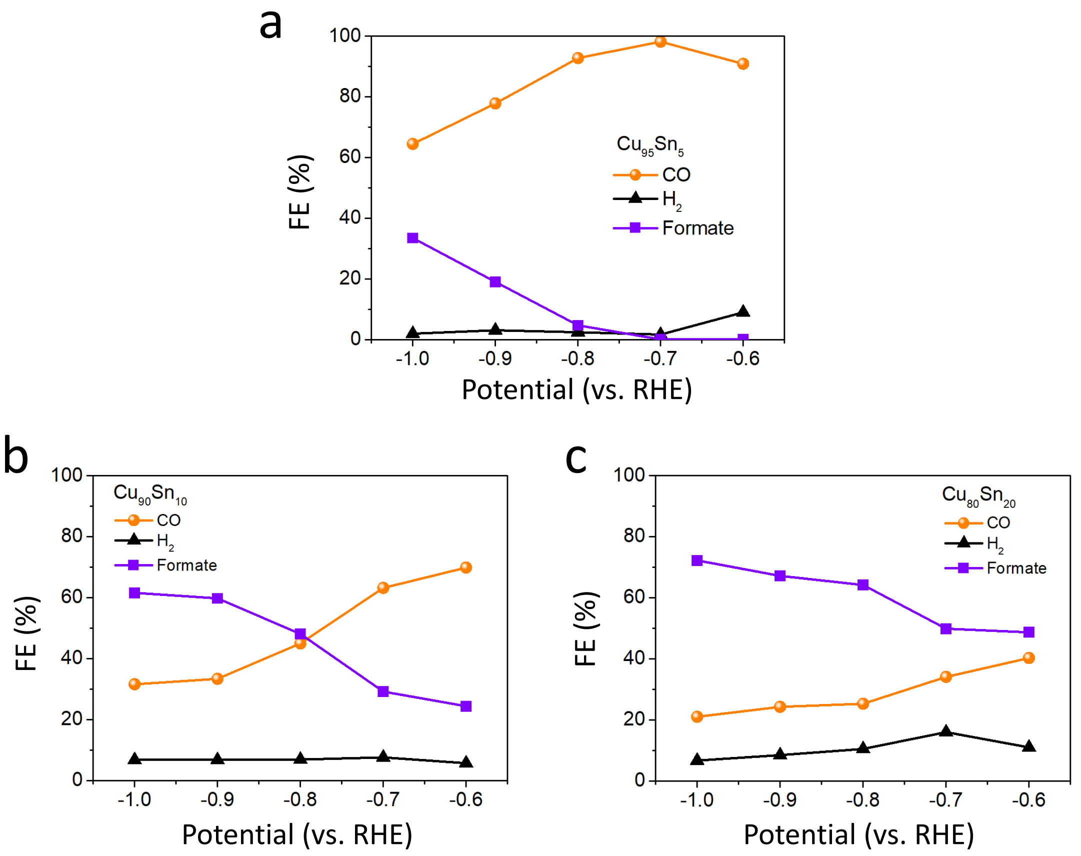

Supplementary Figure 12 | CO_2RR measurements on $Cu_{95}Sn_5$ (a), $Cu_{90}Sn_{10}$ (b), and $Cu_{80}Sn_{20}$ (c). As shown in Supplementary Fig. 12, the $Cu_{95}Sn_5$ exhibits CO dominated reactions from -0.6 to -1.0 V vs RHE, and the corresponding FE_{CO} reaches 98% at -0.7 V. As for $Cu_{90}Sn_{10}$, the FE_{CO} still outperforms $FE_{Formate}$ at low potentials from -0.6 to -0.7 V, and then the production of CO and formate becomes similar at -0.8 V with FE of both products at ~45%. With the further increase of overpotential, the $Cu_{90}Sn_{10}$ produces preferentially formate instead of CO. As for $Cu_{80}Sn_{20}$, the $FE_{Formate}$ exceeds the FE_{CO} in the whole potential range and more approaches to the performance of $Cu_{70}Sn_{30}$.

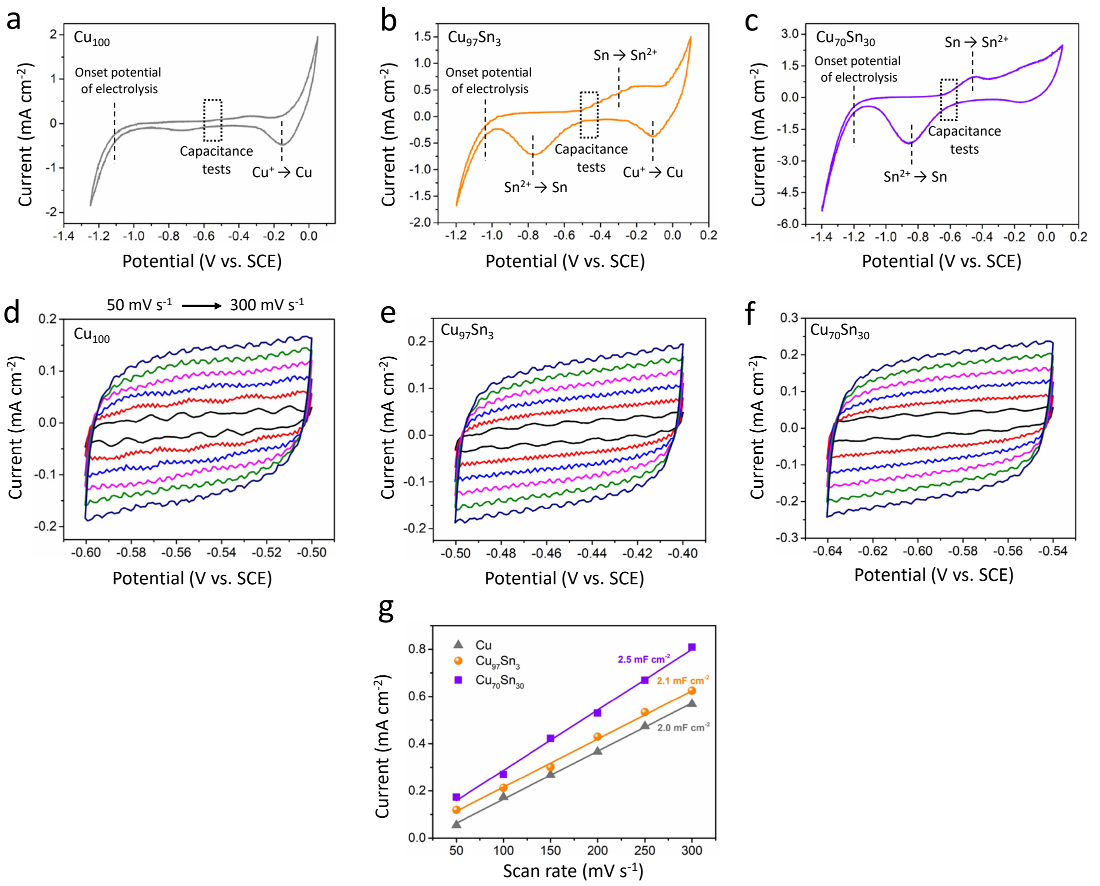

Supplementary Figure 13 | Electrochemically active surface area (ECSA) estimated from the double-layer capacitance. (**a-c**) CV curves for the identification of the capacitance measurements. (**d-f**) CV curves at different scan rates from 50 to 300 mV s^{-1} for Cu_{100}, $Cu_{97}Sn_3$ and $Cu_{70}Sn_{30}$, respectively. (**g**) Double-layer capacitance of different electrodes. The fluctuation in CV curves can be attributed to the CO_2 gas bubbling during the tests and the Autolab setup is very sensitive to the current change. Note that the fitting error in Supplementary Fig. 13g is very small, which suggests reliable double-layer capacitance calculation.

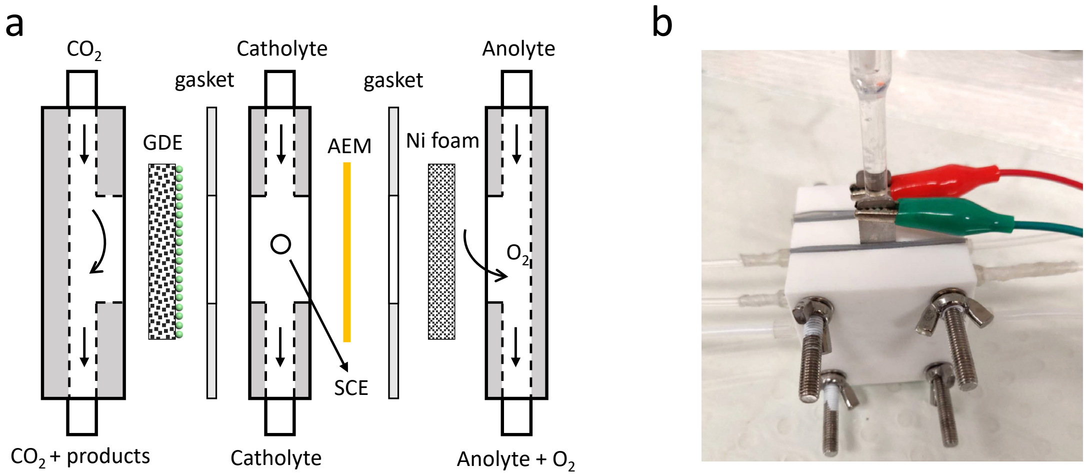

Supplementary Figure 14 | Graphic illustration of a home-customized flow cell.

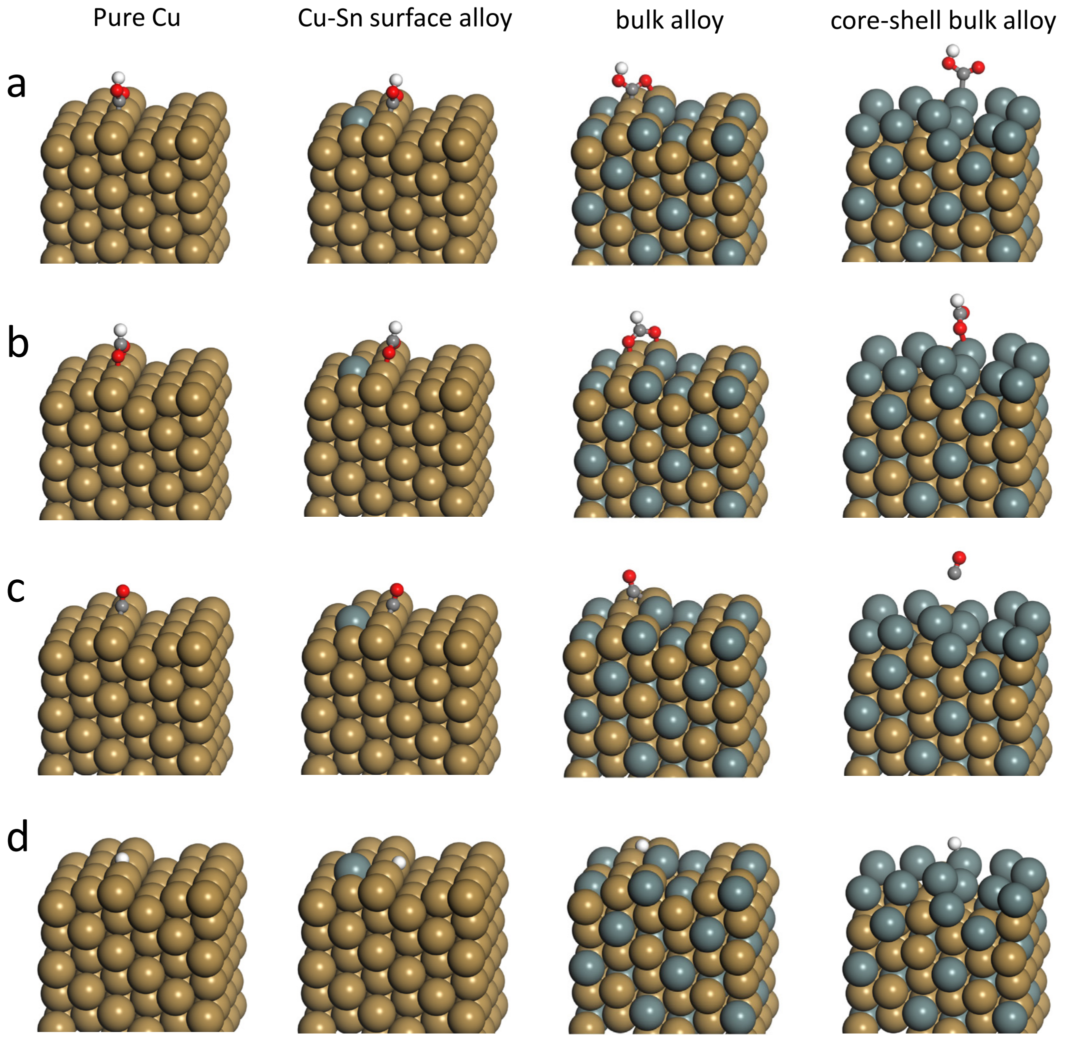

Supplementary Figure 15 | Optimized DFT model with different intermediates. Surface slabs with adsorbed (**a**) COOH*, (**b**) OCHO*, (**c**) CO*, and (**d**) H* on stepped facets of pure Cu, $Cu_{97}Sn_3$ and $Cu_{70}Sn_{30}$. The dark goldenrod, slate grey, grey, red and white balls represent Cu, Sn, C, O and H atoms, respectively.

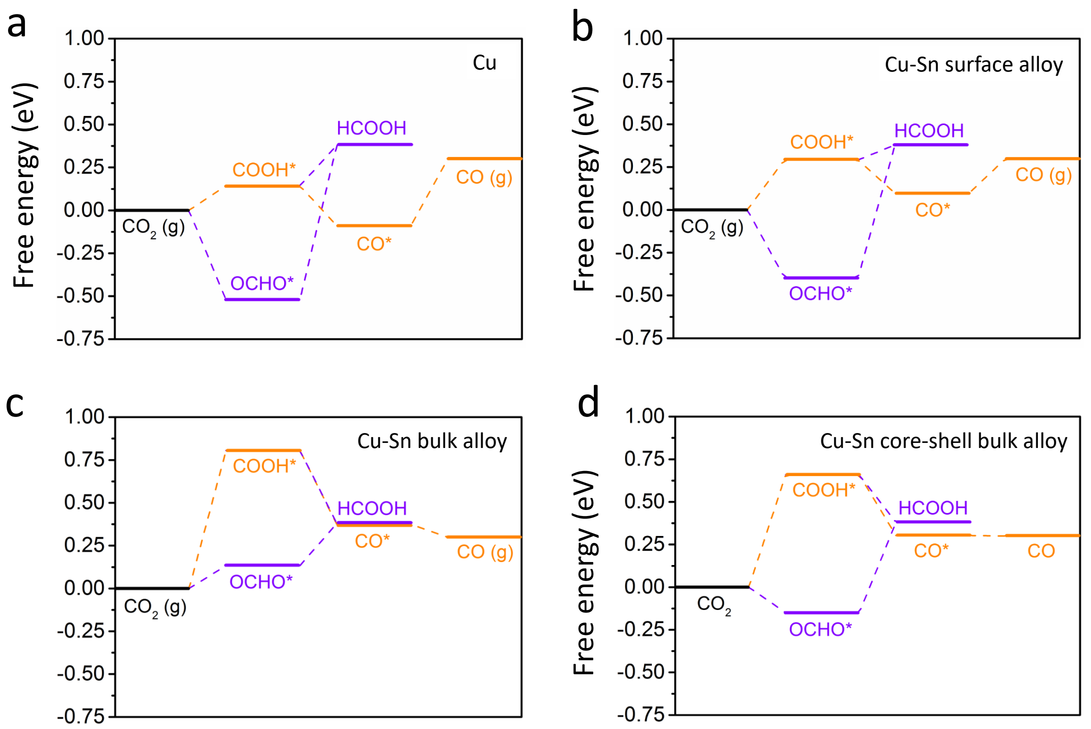

Supplementary Figure 16 | The calculated free energy diagrams for CO_2-to-CO and CO_2-to-HCOOH conversion on four samples.

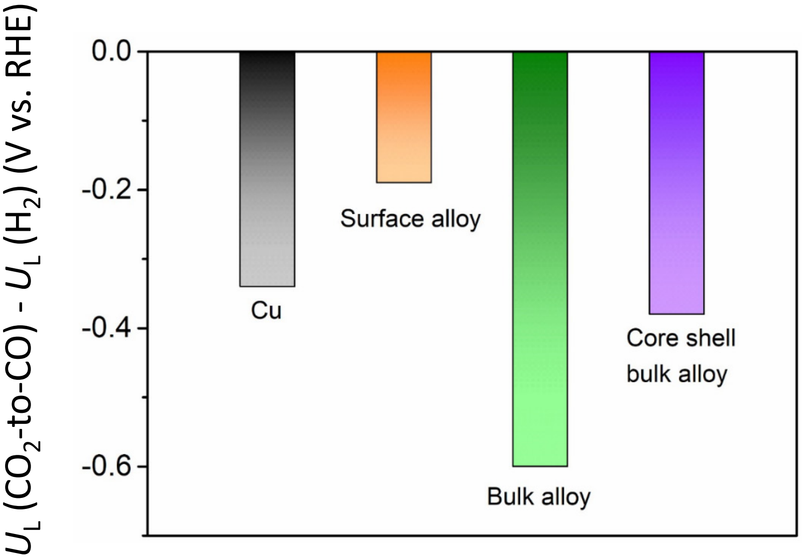

Supplementary Figure 17 | The difference in limiting potentials for CO_2-to-CO reduction and H_2 evolution on the catalysts. Previous studies have shown that the difference between thermodynamic limiting potentials for CO_2RR and HER (denoted as $U_L(CO_2)-U_L(H_2)$, in which U_L is defined as the lowest potential that all reaction steps are downhill in free energy) can reflect the selectivity of CO_2RR. According to this definition, a more positive value indicates a higher selectivity. Figure S17 shows the difference in limiting potentials for four samples, where surface alloy demonstrates a considerably more positive value than other samples, suggesting the greatly enhanced selectivity for CO_2-to-CO conversion.

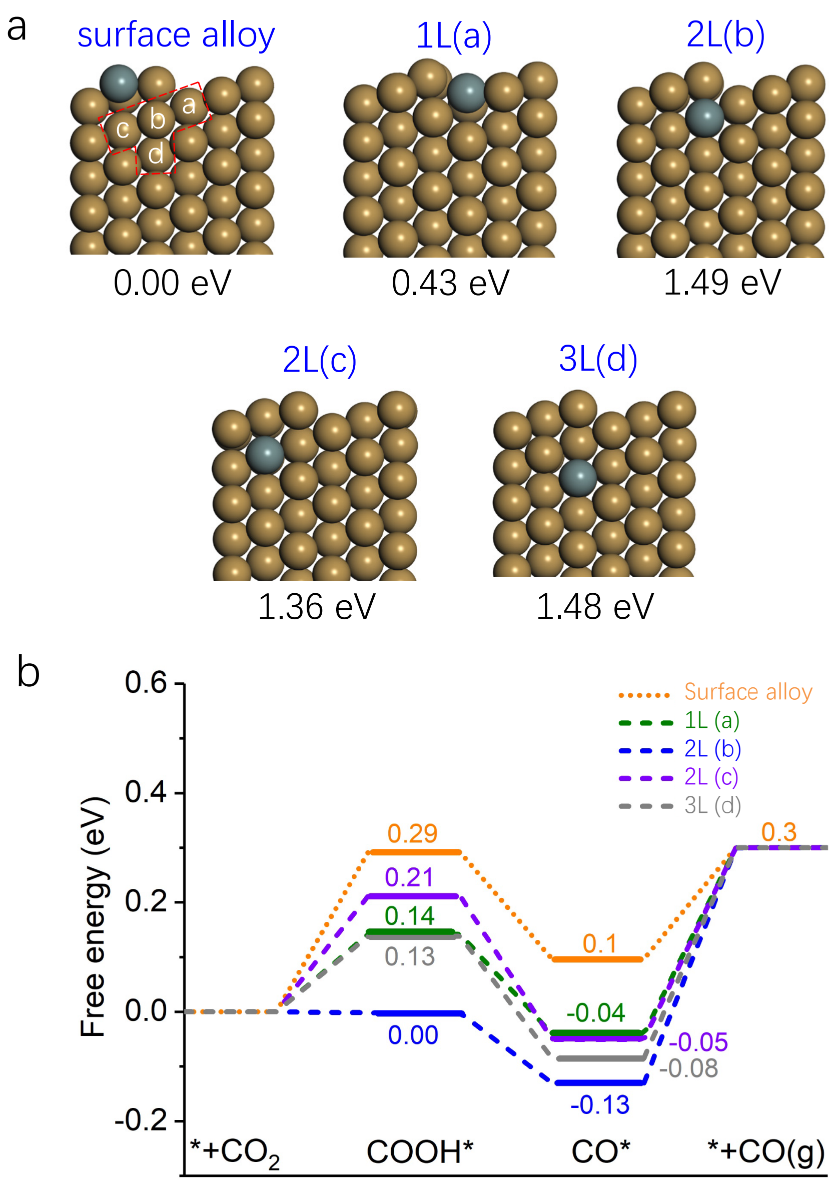

Supplementary Figure 18 | DFT simulations of CO_2RR on Cu-Sn alloys with Sn atoms in different layers. (**a**) The relative substitution energy of a Cu atom by an Sn atom at different positions. All are given in eV. (**b**) The calculated free energy diagrams of CO_2-to-CO conversion for Cu-Sn single-atom alloys with different Sn position.

To understand the surface segregation of Sn atoms from the thermodynamical point of view. New density function theory (DFT) simulations have been carried out to investigate the possible position of Sn atom in single-atom Cu-Sn alloy, i.e. if the Sn prefers to sit on the surface or in the bulk. We calculated the relative substitution energy, which corresponds to the difference between the energy of substitution of a Cu atom in an inner layer and the energy of substitution of a Cu

atom at the surface, as shown in Supplementary Fig. 18a. The results suggest that only sit in the sub-surface layers, the substitution energy barrier of Sn atoms significantly increases to ~1.5 eV for 2L(b) and 3L(d) position. Therefore, the Sn atoms prefer to anchor on the surface of Cu nanoparticles rather than in bulk from an energetics perspective.

To investigate the effects of Sn atoms from different layers, we also calculated the free energy diagrams of CO_2-to-CO conversion for Cu-Sn single-atom alloys with different Sn positions including 1L(a), 2L(b), 2L(c) and 3L(d) (Supplementary Fig. 18b). For Cu-Sn single-atom alloy with Sn at the surface, the overpotential of CO_2 to-CO conversion is very small (0.29 eV). If the Sn atom locates in the second or the third layer, the electrocatalytic performance also displays a certain degree of enhancement compared with pure Cu, however, the overpotentials of CO_2-to-CO conversion significantly increase to 0.34~0.43 V compared with at the surface. These results indicate that the CO_2RR activities of single-atom Cu-Sn alloys are dependent on Sn atom position, and the single-atom Cu-Sn alloy with Sn at the surface shows the highest CO_2RR activity.

Table S1. Inductively coupled plasma optical emission spectroscopy (ICP-OES) analysis. The influence of oxygen is excluded.

Samples	Cu (at%)	Sn (at%)
$Cu_{97}Sn_3$	97.3	2.7
$Cu_{70}Sn_{30}$	72.8	27.2

Table S2. Recently reported Cu-Sn catalysts for CO_2RR in H-cells.

Catalysis	FE_{CO} (%, V vs RHE)	FE_{HCOOH} (%, V vs RHE)	Current density (mA cm^{-2}, V vs RHE)	Ref.
**$Cu_{97}Sn_3$ surface alloy**	**98% at -0.7 V**	**/**	**78 at -1.0 V**	**Our work**
**$Cu_{70}Sn_{30}$ bulk alloy**	**/**	**88.8% at -1.0 V**	**92 at -1.0 V**	**Our work**
CuSn NPs/C-A	~20% at -1.0 V	~70% at -1.0 V	~13 at -1.0 V (HCOOH)	16
$CuSn_3$/carbon paper	/	95% at -0.5 V	31 at -0.5 V (HCOOH)	8
Cu-Sn nanoparticles	/	92% at -0.95 V	9 at -0.95 V (HCOOH)	17
Cu-Sn electrodeposition	/	81% at -1.1 V	13 at -1.1 V (HCOOH)	18
Cu@Sn nanocones	/	90.4% at -1.1 V	57.7 at -1.1 V (HCOOH)	19
7/0.8 Cu/SnO_2	93% at -0.7 V	/	/	20
Sn/Cu-nanofiber	80% at -1.0 V	/	53 at -1.0 V (CO)	21
Cu-Sn dendrite	~90% at-0.7 V	/	11.5 at -1.1 V (CO)	22
Cu-Sn foam	94% at -0.7 V	/	4.7 at -0.8 V (CO)	23
3D-h Cu-Sn	98.6% at -0.45 V	/	4.5 at -0.45 V (CO)	24
OD-Cu-Sn	90% at -0.6 V	/	1.0 at -0.6 V (CO)	6

Table S3. Performance of recently reported CO_2-to-CO electrocatalysts in flow cells.

Catalysis	E (V vs. RHE)	j_{CO} (mA cm^{-2})	FE (%)	Electrolyte	Ref.
$Cu_{97}Sn_3$	-0.45	100	87	1 M KOH	This work
Ni-N/PCFM	-0.7	~115	~80	0.5 M $KHCO_3$	25
Ni-N-C	-0.7	~85	~90	1 M $KHCO_3$	26
Ni-N-C	-0.8	~110	97%	0.5 M $KHCO_3$	27
Fe-N-C	-0.45	94	~90	0.5 M $KHCO_3$	28
CoPc@Fe-N-C	-0.55	~100	>90%	0.5 M KOH	29
CoSA/HCNFs	-0.6	~100	~70	1 M $KHCO_3$	30
CoPc	-0.65	70.5	94	1 M KOH	31
CoPc-N	-0.66	31	94	1 M KOH	32
Ag/PTFE	-1.0	~160	~90	1 M $KHCO_3$	33
	-0.7	~150	~90	1 M KOH	33
Ag/MPL-3C	-0.7	98.5	98.5	0.1 M $KHCO_3$	34
Au	-0.7	~110	/	1 M KOH	35
Porous Zn	-0.9	~100	~67%	1 M $KHCO_3$	36
	-0.62	100	~70%	1 M KOH	36

Table S4. Contribution to the free energy of adsorbates and non-adsorbed gas-phase molecules from ZPT correction, enthalpic temperature correction, and entropy contribution, respectively. All are given in eV.

Species	ZPE	$\int C_P dT$	$-TS$
$COOH^*$	0.62	0.10	-0.18
$OCHO^*$	0.62	0.10	-0.23
CO^*	0.19	0.08	-0.15
CO_2	0.31	0.10	-0.65
CO	0.14	0.09	-0.67
H_2	0.27	0.09	-0.42
H_2O	0.58	0.10	-0.65
HCOOH	0.90	0.11	-1.02

Reference

1 Kresse, G. & Hafner, J. Ab initio molecular-dynamics simulation of the liquid-metal-amorphous-semiconductor transition in germanium. *Phys. Rev. B* **49**, 14251 (1994).

2 Kresse, G. & Joubert, D. From ultrasoft pseudopotentials to the projector augmented-wave method. *Phys. Rev. B* **59**, 1758 (1999).

3 Kresse, G. & Furthmüller, J. Efficient iterative schemes for ab initio total-energy calculations using a plane-wave basis set. *Phys. Rev. B* **54**, 11169 (1996).

4 Perdew, J. P., Burke, K. & Ernzerhof, M. Generalized gradient approximation made simple. *Phys. Rev. Lett.* **77**, 3865 (1996).

5 Grimme, S., Antony, J., Ehrlich, S. & Krieg, H. A consistent and accurate ab initio parametrization of density functional dispersion correction (DFT-D) for the 94 elements H-Pu. *J. Chem. Phys.* **132**, 154104 (2010).

6 Sarfraz, S., Garcia-Esparza, A. T., Jedidi, A., Cavallo, L. & Takanabe, K. Cu-Sn bimetallic catalyst for selective aqueous electroreduction of CO_2 to CO. *ACS Catal.* **6**, 2842-2851 (2016).

7 Monkhorst, H. J. & Pack, J. D. Special points for Brillouin-zone integrations. *Phys. Rev. B* **13**, 5188 (1976).

8 Zheng, X. *et al.* Theory-guided Sn/Cu alloying for efficient CO_2 electroreduction at low overpotentials. *Nat. Catal.* **2**, 55-61 (2019).

9 Peterson, A. A., Abild-Pedersen, F., Studt, F., Rossmeisl, J. & Nørskov, J. K. How copper catalyzes the electroreduction of carbon dioxide into hydrocarbon fuels. *Energy Environ. Sci.* **3**, 1311-1315 (2010).

10 Ju, W. *et al.* Understanding activity and selectivity of metal-nitrogen-doped carbon catalysts for electrochemical reduction of CO_2. *Nat. Commun.* **8**, 944, doi:10.1038/s41467-017-01035-z (2017).

11 Nørskov, J. K. *et al.* Trends in the exchange current for hydrogen evolution. *J. Electrochem. Soc.* **152**, J23-J26 (2005).

12 Thuvander, M. *et al.* Quantitative atom probe analysis of carbides. *Ultramicroscopy* **111**, 604-608 (2011).

13 Felfer, P. *et al.* New approaches to nanoparticle sample fabrication for atom probe tomography. *Ultramicroscopy* **159**, 413-419 (2015).

14 Gault, B., Moody, M. P., Cairney, J. M. & Ringer, S. P. *Atom Probe Microscopy*. Vol. 160 (Springer Science & Business Media, 2012).

15 Nguyen-Phan, T.-D. *et al.* Understanding three-dimensionally interconnected porous oxide-derived copper electrocatalyst for selective carbon dioxide reduction. *J. Mater. Chem. A* **7**, 27576-27584 (2019).

16 Wang, P. *et al.* Phase and structure engineering of copper tin heterostructures for efficient electrochemical carbon dioxide reduction. *Nat. Commun.* **9**, 1-10 (2018).

17 Jiang, X. *et al.* A highly selective tin-copper bimetallic electrocatalyst for the electrochemical reduction of aqueous CO_2 to formate. *Appl. Catal. B: Environ.* **259**, 118040 (2019).

18 Li, M. *et al.* Modulated Sn oxidation states over Cu_2O-derived substrate for selective electrochemical CO_2 reduction. *ACS Appl. Mater. Inter.* **12**, 22760, (2020).

19 Chen, C. *et al.* Sharp Cu@Sn nanocones on Cu foam for highly selective and efficient

electrochemical reduction of CO_2 to formate. *J. Mater. Chem. A* **6**, 19621-19630 (2018).

20 Li, Q. *et al.* Tuning Sn-catalysis for electrochemical reduction of CO_2 to CO via the core/shell Cu/SnO_2 structure. *J. Am. Chem. Soc.* **139**, 4290-4293 (2017).

21 Ju, W. *et al.* Electrocatalytic Reduction of Gaseous CO_2 to CO on Sn/Cu-Nanofiber-Based Gas Diffusion Electrodes. *Adv. Energy Mater.* **9**, 1901514 (2019).

22 Ju, W. *et al.* Sn-decorated Cu for Selective Electrochemical CO_2 to CO Conversion: Precision Architecture beyond Composition Design. *ACS Appl. Energy Mater.* **2**, 867-872 (2018).

23 Zeng, J. *et al.* Advanced Cu-Sn foam for selectively converting CO_2 to CO in aqueous solution. *Appl. Catal. B: Environ.* **236**, 475-482 (2018).

24 Yoo, C. J. *et al.* Compositional and Geometrical Effects of Bimetallic Cu-Sn Catalysts on Selective Electrochemical CO_2 Reduction to CO. *ACS Appl. Energy Mater.* (2020).

25 Yang, H. *et al.* Carbon dioxide electroreduction on single-atom nickel decorated carbon membranes with industry compatible current densities. *Nat. Commun.* **11**, 1-8 (2020).

26 Möller, T. *et al.* Efficient CO_2 to CO electrolysis on solid Ni-N-C catalysts at industrial current densities. *Energy Environ. Sci.* **12**, 640-647 (2019).

27 Zhang, T. *et al.* Nickel-Nitrogen-Carbon Molecular Catalysts for High Rate CO_2 Electro-reduction to CO: On the Role of Carbon Substrate and Reaction Chemistry. *ACS Appl. Energy Mater.* **3**, 1617-1626 (2020).

28 Gu, J., Hsu, C.-S., Bai, L., Chen, H. M. & Hu, X. Atomically dispersed Fe^{3+} sites catalyze efficient CO_2 electroreduction to CO. *Science* **364**, 1091-1094 (2019).

29 Lin, L. *et al.* Synergistic Catalysis over Iron-Nitrogen Sites Anchored with Cobalt Phthalocyanine for Efficient CO_2 Electroreduction. *Adv. Mater.* **31**, 1903470 (2019).

30 Yang, H. *et al.* Highly efficient utilization of single atoms via constructing 3D and free-standing electrodes for CO_2 reduction with ultrahigh current density. *Nano Energy* **70**, 104454 (2020).

31 Wang, M. *et al.* CO_2 electrochemical catalytic reduction with a highly active cobalt phthalocyanine. *Nature Commun.* **10**, 1-8 (2019).

32 Lu, X. *et al.* High-performance electrochemical CO_2 reduction cells based on non-noble metal catalysts. *ACS Energy Lett.* **3**, 2527-2532 (2018).

33 Dinh, C.-T., García de Arquer, F. P., Sinton, D. & Sargent, E. H. High rate, selective, and stable electroreduction of CO_2 to CO in basic and neutral media. *ACS Energy Lett.* **3**, 2835-2840 (2018).

34 Wang, R. *et al.* Maximizing Ag utilization in high-rate CO_2 electrochemical reduction with a coordination polymer-mediated gas diffusion electrode. *ACS Energy Lett.* **4**, 2024-2031 (2019).

35 Verma, S. *et al.* Insights into the low overpotential electroreduction of CO_2 to CO on a supported gold catalyst in an alkaline flow electrolyzer. *ACS Energy Lett.* **3**, 193-198 (2017).

36 Luo, W., Zhang, J., Li, M. & Züttel, A. Boosting CO production in electrocatalytic CO_2 reduction on highly porous Zn catalysts. *ACS Catal.* **9**, 3783-3791 (2019).
