# Supplementary material for: Isolated copper–tin atomic interfaces tuning electrocatalytic CO2 conversion
Source: Nat Commun. 2021 Mar 4;12:1449. doi: 10.1038/s41467-021-21750-y (PMC7933149; doi:10.1038/s41467-021-21750-y)
Supplement: Supplementary file 4 — Description of Additional Supplementary Files [file 41467_2021_21750_MOESM4_ESM.pdf]

Description of Additional Supplementary Files

Title: Supplementary Movie 1.

Description: 3D tomography of the $Cu_{97}Sn_3$ nanoparticle demonstrates the position relationship of Sn dopants (green) and Cu matrix (purple).
